# The order of events on *Avian coronavirus* life cycle shapes the order of quasispecies evolution during host switches

**DOI:** 10.1590/1678-4685-GMB-2022-0034

**Published:** 2022-06-06

**Authors:** Paulo E. Brandão, Mikael Berg, Beatriz A. Leite, Sheila O.S. Silva, Sueli A. Taniwaki

**Affiliations:** 1 Universidade de São Paulo, Faculdade de Medicina Veterinária e Zootecnia, Departamento de Medicina Veterinária Preventiva e Saúde Animal, São Paulo, SP, Brazil.; 2 Swedish University of Agricultural Sciences, Department of Biomedical Sciences and Veterinary Public Health, Section of Virology, Uppsala, Sweden.

**Keywords:** Coronavirus, quasispecies, cell culture, spillover

## Abstract

To understand the population genetics events during coronavirus host switches, the Beaudette strain of *Avian coronavirus* (AvCoV) adapted to BHK-21 cells was passaged 15 times in VERO cells, the virus load and the variants at each passage being determined by RT-qPCR and genome-length deep sequencing. From BHK-21 P2 to VERO P3, a trend for the extinction of variants was followed by stability up to VERO P11 and both the emergence and the rise in frequency in some variants, while the virus loads were stable up to VERO P12. At the spillover from BHK-21 to VERO cells, variants that both emerged, showed a rise in frequency or were extinguished were detected on the spike, while variants at the M gene showed the same pattern only at VERO passage 13. Furthermore, nsps 3-5, 9 and 15 variants were detected at lower passages compared to the consensus sequences, with those at nsp3 being detected in the spectra also at higher passages. This suggests that quasispecies coronavirus evolution in spillovers follows the virus life cycle, starting with the evolution of the receptor binding proteins, followed by the replicase and then proteins involved in virion assembly, keeping the general fitness of the mutant spectrum stable.

## Introduction

*Avian coronavirus* (AvCoV) (*Nidovirales*: *Coronaviridae*: *Coronavirinae*: *Gammacoronavirus*), for which chickens (*Gallus gallus*) are the natural hosts, can be isolated in chicken embryo eggs but is of a rather fastidious growth in cell cultures (Cook et al., 2012), with Beaudette being the more easily cultivated strain in avian and mammalian cell lines.

AvCoV presents an enveloped pleomorphic virion of approximately 120 nm in diameter, and the genome is a positive-sense single-stranded RNA of 27.6 kb with a 5’ cap and a poly-A tail. It encodes 23 proteins, with a nontranslated region (UTR) at the 5’ and 3’ ends of approximately 500 nt each; all but the N gene are structurally poly but functionally monocistronic and include a leader sequence at the 5’ end with transcription regulating sequences (TRS) complementary to the leader upstream of each ORF (Masters, 2006; Cavanagh, 2007).

The 5’ two-thirds of the genome encode the replicase polyprotein that gives rise to 15 nonstructural proteins (nsps 2 to 16, as nsp1 is absent in AvCoV) via proteolytic cleavage by nsps 2 (picornavirus 3C-like protease) and 3 (papain-like protease). Those 15 proteins, including nsp12, the RNA-dependent RNA-polymerase, and nsp14, a 3’-5’ exonuclease, are involved in the replication and transcription steps (Ziebuhr and Snijder, 2007).

On the envelope, the spike protein, with 1,163 aa, is found in a trimeric form, with 20 nm projections above the surface of the envelope; each monomer is composed of the S1 amino-terminal external (with the receptor binding site) and the S2 carboxi-terminal internal (with the fusogenic motif) domains (Masters, 2006).

Downstream of the S gene, one finds gene E (99 aa), which codes for the envelope protein, an essential protein for the assembly of virions, together with the M (membrane) protein (223 aa) that comes next on the genome order. The most 3’ gene codes for the nucleocapsid N protein (409 aa), an RNA-binding phosphoprotein that makes the coronavirus helical nucleocapsid (Masters, 2006). Further accessory proteins are encoded in ORFs 3 (proteins 3a and 3b) and 5 (proteins 5a and 5b) with possibly nonessential functions in virus replication (Casais et al., 2005; Britton et al., 2006).

AvCoV readily replicates on the respiratory, reproductive and enteric tracts as well as in the kidneys of chickens but has also been found to have switched hosts to wild birds and other domestic species, such as turkeys and quail (Cavanagh, 2007; Torres et al., 2017, Villarreal et al., 2007a,b). Host switches from bats and rodents with intermediate hosts are also proposed as the origin of human coronaviruses HCoV-NL63, HCoV-229E, HCoV-OC43, HCoV-HKU1, MERS-CoV, SARS-CoV-1 and SARS-CoV-2 (Cui et al., 2019; Andersen et al., 2020).

Due to its role in receptor binding, the spike protein is considered the main vector in coronavirus interspecies jumps, as it overcomes the receptor diversity barrier among different host species (Lu et al., 2015). Nonetheless, a more systematic comprehension of coronavirus host switches is hampered by the almost exclusive focus on the spike protein and the scarcity of host and virus models suitable for experimental evolution studies.

To overcome these limitations, this manuscript reports the complete genome quasispecies evolution of AvCoV after an artificial spillover from BHK-21 (baby hamster kidney) to VERO (African green monkey) cell lines as an experimental evolution model to help uncover coronavirus population genetic features that could be markers of interspecies jumps.

## Material and Methods

### Viruses and cells

The Beaudette strain of AvCoV lineage GI-1 at the second passage in BHK-21 cells (baby hamsters kidney cells from Golden Syrian hamsters *Mesocricetus auratus*, monlolayyer passage number=110) with a virus load of 9.50E+06 genome copies/µL was used as the ancestor virus. VERO cells (renal cells from African green monkey *Chlorocebus* sp, monolayers passages numbers 153 to 164) were purchased from the Adolfo Lutz Institute, Brazil and maintained in MEM with 10% fetal calf serum (FCS) at 37 ºC.

### Passages of the ancestral virus in Vero cells

The Beaudette strain was passaged 15 times in VERO cells starting with the ancestor virus in BHK-21 cells with 9.50E+06 genome copies/µL, using a fixed volume instead of a fixed virus load between passages to assess the effects of the quasispecies evolution on virus loads, which is considered a measurement of virus fitness, and to mimic the condition found in natural transmission of coronaviruses.

The ancestor virus was thawed in an ice bath and centrifuged at 1,000 g/4 °C for 10 minutes, and the supernatant was used as an inoculum. The growth medium was removed from 100% confluent, 48 h-old VERO cell monolayers in 25 cm2 flasks. The monolayers were then washed with FCS-free MEM, and 1 ml of the inoculum was added to the monolayer. In parallel, another VERO monolayer was mock-inoculated with 1 mL of FBS-free MEM to serve as a control. After incubation for 1 h at 37 °C, the inocula were discarded, and the cells were incubated for 48 h at 37 °C and monitored for coronavirus syncytial cytopathic effect (CPE) under 100x magnification in a light microscope. The monolayers were then frozen at -80 °C, and the same procedures were used for the following passages.

### Real-time quantitative PCR (qPCR) of AvCoV genomic RNA

Genome copy numbers in each passage, in the ancestor virus, and in the mock passages were determined by qPCR as described by Callison et al. (2006) (in triplicate) using β-actin as an endogenous control (IDT Integrated DNA Technologies proprietary primers 5’ACAGAGCCTCGCCTTTG3’/5’CCTTGCACATGCCGGAG3’) (in duplicate) with a Power SYBR® Green RNA-to-Ct™ 1-Step kit (Applied Biosystems) after total RNA extraction with RNeasy® Mini kit (Qiagen). All qPCRs were carried out in a 7500 Real-Time PCR system (Applied Biosystems) (48 °C/30 min; 95 °C/10 min; 45 cycles of 95 °C/15 s and 60 °C/40 s) and melting curve analysis was performed. Absolute quantification of AvCoV genomes was derived from a dilution standard curve ranging from 10^3^ to 10^9^ copies per reaction (slope=-3.536; y-intercept=43.665) normalized to β-actin.

### Deep sequencing

Total RNA was extracted using TRIzol® LS (Ambion) and RNeasy® Mini kit (Qiagen) after supernatants from the ancestor virus and from the 15 passages in VERO cells were filtered through 0.45 µm filters and treated with Turbo™ DNase (Ambion) and RNase™ Cocktail Enzyme mix (Ambion) per the manufacturer’s instructions.

Next, ds-cDNAs were prepared using random primers, SuperScript™ III Reverse Transcriptase (Invitrogen) and 3’-5’ exo - Klenow DNA Polymerase™ (Ambion) per the manufacturer’s instructions, and libraries were obtained using Nextera® Sample Preparation (Illumina). The ds-cDNAs were then purified with magnetic beads using AMPure® XP (Beckman Coulter Life Sciences) and quantified with qPCR using KAPA® Fast Universal (Sigma-Aldrich)*.* Reads were then obtained in a NextSeq500 MID Output 300 System (Illumina) (2 x 150 bp).

### Mutant spectra analysis

The dominant consensus genome for the ancestral virus was obtained by the reference assembly in CLC Genomics Workbench 20 (Qiagen), and the complete genome of the Beaudette strain (NC_001451.1) was used as a reference. Then, the genome of each previous passage was used as a reference to assemble the genome of the next passage up to passage 15 in VERO cells.

Variants were detected after reads were mapped again using the consensus obtained for each specific passage using the following parameters in CLC: low frequency variants detection, 100 coverage, 10 counts, 5% frequency, 1% significance and 20 central and neighborhood qualities.

### Codon usage analysis

The codon adaptation index (CAI) was calculated for the consensus sequences of the ancestor virus and the 15 VERO passages based on *Mesocricetus auratus* (Syrian hamster, origin of the BHK-21 lineage) and *Chlorocebus sabaeus* (the closest species for which codon usage data was available regarding the origin of the VERO lineage) codon usage tables available at the Kazusa Codon Usage Database (https://www.kazusa.or.jp/codon/) and calculated with CalCAlc2 (Puigbò *et al*., 2008).

## Results

### Passages in VERO cells and virus loads

Syncytial CPE in all passages was evident at 24 h postinoculation (hpi) and reached a maximum at 48 hpi, with no noticeable differences in CPE intensity among passages. All mock-infected monolayers showed no CPE.

The viral load in the ancestor virus was 9.50E+06 copies/µL sample and 7.61E+06 copies/µL at VERO P1; the average viral load for the VERO passages was 4.49E+07 copies/µL sample, with maximum and minimum viral loads at VERO passages 12 (2.18E+08 copies/µL sample) and 13 (3.81E+06 copies/µL sample), respectively (Table 1). All mock-infected monolayers showed no detectable AvCoV.

**Table 1 -:** Genome size and coverage, virus load (genome copy number), total number (#) of variants after passages of the Beaudette strain of AvCoV in BHK-21 and VERO cells and the frequency of non-synonymous (ns) variants.

Passage	Genome size	Coverage	Viral load (Copies/µL sample)	# variants/ # of ns variants (% ns variants)
BHK-21 P2	27,607	93,920	9.50E+06	51/31 (60.8)
VERO P1	27,608	72,071	7.61E+06	32/13 (40.6)
VERO P2	27,608	63,650	5.68E+06	34/15 (44.1)
VERO P3	27,608	81,190	6.53E+06	12/2 (16.7)
VERO P4	27,608	83,073	1.20E+07	15/5 (33.3)
VERO P5	27,608	102,456	3.72E+07	18/4 (22.2)
VERO P6	27,602	131,34	3.46E+07	16/4 (25)
VERO P7	27,602	107,323	1.36E+07	16/3 (18.8)
VERO P8	27,602	123,427	4.30E+07	10/0 (0)
VERO P9	27,602	108,64	8.90E+07	14/0 (0)
VERO P10	27,602	111,722	7.50E+07	16/0 (0)
VERO P11	27,602	108,441	3.80E+07	18/2 (11.1)
VERO P12	27,602	47,636	2.18E+08	50/10 (20)
VERO P13	27,602	47,286	3.81E+06	56/19 (33.9)
VERO P14	27,602	15,629	3.51E+07	46/14 (30.4)
VERO P15	27,602	72,795	5.44E+07	40/11 (27.5)

### Mutant spectra analysis

Fifty-one variants were found in the ancestor virus; the minimum and maximum number of variants were reached at VERO passages 8 (n=10) and 13 (n=56), respectively (Table 1). A low correlation was found between the virus load and the number of variants (r^2^=0.58) and the coverage and the number of variants (r^2^=0.03), including the data from the ancestor virus.

Considering all VERO passages and the ancestor virus, variants were found in all regions except nsp11, 3b and 5b, with a total of 209 variants.

All reads were deposited in the GenBank SRA database under accession number PRJNA736341. The genome of the Beaudette strain at VERO passage 15 was deposited under the accession # MZ368698. The other full genomes were not submitted to GenBank to avoid redundancy.

### Variants found at the spillover

The total number of variants dropped from 51 in the ancestor virus to 32 in VERO P1 (Table 1). Nonsynonymous mutations were found for most proteins except nsps 7-8, 10-11, 15, 3a-b and 5a-b, and the highest frequencies were at ORF X (2%) and the spike glycoprotein (1.03%). There were 12 nonsynonymous mutations in the spike glycoprotein (S25P, V27G, S53F, S53Y, T84M, K116R, F469S, S656F, S969A, V1013I, K1015E and K1015N) and one nonsynonymous mutation (C35S) at ORF X.

No mutations were found for the dominant consensus between the ancestor virus and VERO P1 except a 1-nt shorter 5’UTR for the former.

### Variants for the VERO passages

**Figure 1-:**
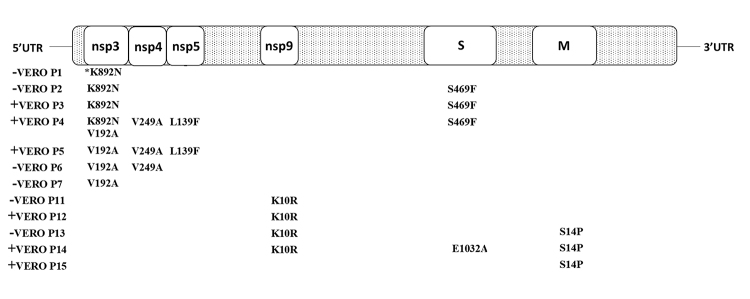
A cartoon representation of *Avian coronavirus* genome with the genes (not in scale) showing the positions and the amino acid states (to the right of the position numbering) found in variants regarding the dominant amino acids states (to the left the position numbering) after 15 passages of the Beaudette strain in VERO cells. - =decrease in virus load; + = rise in virus load; *= also found in the ancestor virus (BHK-21 2^nd^ passage).

To focus on the nonsynonymous variants with a possible influence on virus fitness, the frequency of variants resulting in amino acid changes whose frequency oscillated between passages (Table 2 and Figure 1) was calculated with regards to the total length of the corresponding gene for those passages with a fluctuation in virus loads of at least one order of magnitude (E1): VERO P12 to P13 (drop in virus load) and VERO P3 to P4, P11 to P12 and P13 to P14 (increase in virus load).

**Table 2 -:** Nucleotide mutations and amino acids changes detected after passages of the Beaudette strain of AvCoV in BHK-21 and VERO cells, showing the passage (P) they were detected at in both the consensus and the consensus levels; syn=synonymous mutation.

Gene	Nucleotide mutation/ amino acid change	Detection in consensus sequence	Detection in subconsensus sequence
nsp3	G2676T (K892N)	VERO P1 to P2	BKH21 2P to VERO P4
	T575C (V192A)	VERO P5 to P6	VERO P4 to P7
nsp4	T746C (V249A)	VERO P5 to P6	VERO P4 to P6
nsp5	C415T (L139F)	VERO P5 to P6	VERO P4 to P5
nsp8	del 611-616 AGGTTG (del 204-205 KV)	VERO P5 to P6	VERO P5
nsp9	A29G (K10R)	VERO P14 to P15	VERO P11 to P14
nsp15	A702G (syn)	VERO P13 to P14	VERO P1 to P13
S	C1406T (S469F)	VERO P1 to P2	VERO P2 to P4
	A3095C (E1032A)	VERO P13 to P14	VERO P14
M	T40C (S14P)	VERO P13 to P14	VERO P13 to P15

The fluctuation in the frequency of nonsynonymous variants for the membrane protein gene showed the highest (0.82) correlation coefficient with fluctuations in virus loads, while for all other proteins, this value ranged from 0 (S and E) to 0.63 (nsps13 and 16).

Out of the 31 variants with fluctuations in the frequency of nonsynonymous variants that make up these classes, nsp9 K10R, nsp12 H138P, nsp5 A140S and M P14S showed a strong effect size (r^2^=1-0.71) on fluctuations in virus loads, with both an increase in loads and a recovery after the drop in load at VERO P13.

A moderate effect size on virus load (r^2^=0.52) was found for the variants S S469F, nsp5 L139F, nsp3 K892N and nsp3 V192A when the virus load rose from 6.8 to 7.1 log/µL sample from VERO P3 to P4. The remaining 23 variants showed weak or very weak effects on virus loads.

Regarding the dominant consensus sequences for each passage, mutations were detected only for nsps 3-5, 8-9, 15, spike and membrane protein genes. A total of 10 mutations, with only one synonymous mutation, were found from VERO P1 to P15, with four of these between passages 5 and 6 and M S14P being the only mutation detected at the drop in titer in VERO P13. A 6 nt/8 aa (611-616 AGGTTGA/204-205 KV) deletion was found for VERO P6 to P15.

At the 5’UTR, dominant consensus sequences showed a C2A mutation at P14 that reverted to C at P15, and a G11T mutation at P13 and P14 that reverted to G at P15. Also, a A501T mutation at VERO P13 and 14 reverted to A at P15, a A503T mutation at P13 reverted to A at P14, a G504C mutation at P13 and P14 mutated to A at P15 and a C505T mutation at P12, 13 and 14 mutated to A at P15.

### Codon usage analysis

Mean CAI values were stable for the ancestor virus and all VERO passages: 0.61 for the hamster reference and 0.47 for the green monkey reference.

## Discussion

The receptor-binding domain for the AvCoV M41 strain, which is homologous to the Beaudette strain, has been mapped to the 253 N-terminal amino acids of the spike protein, with residues 19-69 being the more critical ones (Promkuntod et al., 2013). Herein, at the spillover (*i.e.*, host change) from BHK-21 to VERO cells, variants emerged (S25P and S53Y) and showed both a rise in frequency (V27G and S53F) or, on the other hand, quenching (T84M) or a decrease in frequency (K116R) in this region of the spike.

α2,3-linked sialic acid has been shown to be a receptor for AvCoV, but the ability of the Beaudette strain to replicate in cell cultures might be due to binding to heparan sulfate via spike amino acids SRRKRS (residues 686-691) (Winter et al., 2006; Madu et al., 2007), which was found to be conserved for all passages in this study. HSP70 has also been suggested to be a part of the receptor for AvCoV in kidney cells (Zhang et al., 2017), which is of specific interest, as BHK-21 and VERO cells are of kidney origin.

BHK-21 and VERO cells have long been reported as susceptible and permissive to the Beaudette strain of AvCoV, and, though BHK-21 cells are refractory, for instance, to SARS-CoV-2, as they do not express the ACE2 cell membrane viral receptor, golden Syrian hamsters, the animals these cells are derived from, are susceptible to this virus, as well *Chlorocebus* sp monkeys, the origin of VERO cells (Otsuki et al., 1979; Cavanagh et al., 1986; Schmitt et al., 2020; Sia et al., 2020; Wang et al., 2020). Thus, though derived from distantly related mammals such as rodents and primates, the two cell lines used in this investigation provide a dependable *in vitro* model for *Avian coronavirus*. 

From the ancestor virus up to VERO P3, a trend for the extinction of variants was observed, what might be a signal of a bottleneck effect, and this was followed by stability up to VERO P11 and both the emergence and the rise in frequency in some variants, while the virus loads were stable up to VERO P12.

It is noteworthy that all mutations in nsps 3-5, 9 and 15 (Table 2 and Figure 1) were detected in the mutant spectra at lower passages compared to the consensus sequences, with those at nsp3 still being detected in the spectra at higher passages. On the other hand, S469F in S and S14P in M were still detected in subconsensus sequences in the mutant spectra at higher passages (Table 2 and Figure 1) compared to their detection in the consensus sequences.

Virions with spikes presenting higher affinity for VERO cells at passage 1 were thus subjected to a second round of selection based on the replicase during the following VERO passages, with amino acid changes at passage 6 in nsps 3-5, 8-9, and 15. These proteins play a role in cotranslational nsps cleavage, compartmentalization of the cytoplasm for virus replication, virus release and RNA transcription (Ziebuhr and Snijder, 2007).

At passage 13, an amino acid change was also detected in the M protein (S14P). M plays a major role in the late phase of the coronavirus cycle, interacting with the envelope E protein during the assembly of new virions (Vennema et al., 1996).

Variants nsp9 K10R, nsp12 H138P, nsp5 A140S and M P14S showed a strong effect size (r^2^ 1-0.71) on virus load increase and may indicate a selective advantage for the mutant spectra at the passages in which they emerged and might have contributed to more efficient RNA synthesis in the case of nsps and more efficient virion assembly in the case of the M protein.

A variant analysis of an Arkansas-type vaccine strain of AvCoV using Illumina technology showed that the spike gene reached population homogeneity, and protection against homologous challenge, *i.e.*, the same virus type for vaccination and challenge, was observed in birds through a study of passages in chicken embryo cells (CEK) (Zegpi et al., 2017), although a restricted (n=7) passage number was used.

The Beaudette strain has also been used to assess the evolution of mutant spectra in serial (up to 65 passages) passages in VERO cells (Fang et al., 2005). Population homogeneity was seen at passage 7, and minor variants were detected afterward, although a much higher passage number was used compared to Zegpi’s et al. study. The limitation in these cases was the use of Sanger sequencing of PCR products, which can result in a rather shallow sequencing when quasispecies is the goal.

No codon usage adaptation was detected either at the spillover or during the VERO passages, an indication that selection based on amino acid changes was the unique vector of AvCoV evolution in VERO cells.

From these results, a pattern can be drawn in regard to the order of emergence and extinction of variants and dominant genomes of AvCoV during adaptation to a new host following the virus life cycle. This starts with the evolution of the receptor binding proteins, followed by the replicase and then proteins involved in virion assembly, keeping the general fitness of the mutant spectrum stable.

Nonetheless, experiments with higher passage numbers and a greater variety of cell lines must be performed to test the validity of this pattern in independent studies. In addition, other methods, such as plaque assays and AvCoV haplotype assembly, need to be used to assess virus fitness.
